# Speech Data for Improved Audiological Evaluation in the Romani Language

**DOI:** 10.3390/audiolres16030065

**Published:** 2026-04-28

**Authors:** Eva Kiktová, Július Zimmermann

**Affiliations:** Language Information and Communication Laboratory, Faculty of Arts, Pavol Jozef Šafárik University in Košice, Šrobárova 2, 041 80 Košice, Slovakia; julius.zimmermann@upjs.sk

**Keywords:** speech, audiology, comprehension, screening, Romani language

## Abstract

Background: This paper describes the development of speech materials in the Romani language intended for audiological and comprehension assessment of Romani-speaking children and adults living in Slovakia. The work responds to the documented lack of linguistic resources and test stimuli in Romani, which limits the accuracy of speech perception and comprehension testing. Method: The existing state of communication-assessment tests used in Slovakia was reviewed, and new Romani-language materials for audiology and comprehension testing were created. The work focused on developing word lists, matrix-based sentence tests, and comprehension sentences that were linguistically verified by native Romani speakers. Results: A set of Romani speech stimuli was developed, including a ten-word screening list, a 50-word illustrated set for pediatric audiometry, an adaptive matrix sentence test for advanced assessment, and a collection of comprehension sentences targeting various linguistic structures. Conclusions: The newly created Romani-language test materials address the absence of suitable diagnostic tools in Slovakia and provide culturally and linguistically appropriate resources for more accurate audiological and comprehension assessment.

## 1. Introduction

It is estimated that around 500,000 Roma live in Slovakia [1,2]. The majority of them are concentrated in the eastern and southern parts of the country. The most famous locations include the Košice and Prešov regions, which are located in eastern Slovakia, followed by the southern areas of the Banská Bystrica and Nitra regions. The Eastern Slovak dialect, similarly to the Central Slovak dialect, forms the basis of the commonly used variety of the language. The potential for using the Romani language is therefore high in these parts of the country. The occurrence of communication disorders does not bypass the Roma population. The often different way of life in socially excluded Roma communities limits their future opportunities in education and subsequently in working life. It also affects their experience, as well as their social and economic development. One of the fundamental prerequisites for a fulfilling life is the manner and quality of communication. The development of communication skills (listening, comprehension, and speech production) represents important milestones in every person’s life.

Human beings naturally communicate through speech, which plays an irreplaceable role in the perceptual processing of information. Therefore, when assessing auditory competencies, it is essential to conduct the examination using speech stimuli. Standard pure-tone audiometry (PTA) is often insufficient, as it focuses only on a narrow range of tones at specific frequencies rather than on the mixture of diverse frequencies (tones and noise) that are contained in speech. Significant PTA results, therefore, do not automatically indicate equally solid outcomes in speech audiometry. Nevertheless, PTA represents a quick and simple method for measuring hearing abilities. However, speech tests are increasingly gaining prominence because their outcomes show greater correspondence with real-life hearing performance in everyday communication situations. They help patients understand how much they can rely on their hearing. It is also important to note that real communicative situations always occur in the presence of more or less prominent background noise, which limits the level of auditory perception.

Despite the availability of Slovak speech audiometry materials, no validated or systematically developed Romani-language speech materials exist, which makes it impossible to accurately assess auditory and comprehension abilities in Romani-speaking individuals. This article introduces a database of suitable stimuli for hearing screening, audiological assessment, and comprehension testing in the Romani language. We believe that the created sets of stimuli will help professionals overcome communication barriers caused by the use of different languages, either in everyday interactions or directly during assessments, through the use of stimuli that are natural for the patient.

## 2. Related Works

This section summarizes existing speech audiometry tools used in Slovakia and highlights their limitations for Romani-speaking children and adults.

In Slovakia, 10 sets of 10 words each, designed in 1975, are used for hearing assessment. Each set includes one to three-syllable words. A more recent Slovak speech audiometry test for children enables identification of the presented word using pictures and comprises 80 words divided into eight sets [3]. Both of these tests contain nouns, adjectives, verbs, and adverbs. These materials are available exclusively in Slovak and do not always reflect the vocabulary typically used by Romani-speaking children. A Slovak 10-word screening set suitable for rapid testing is currently under development.

The level of speech comprehension is assessed using various diagnostic testing tools. However, many of them are not adapted to the Slovak language and are therefore used in the Slovak Republic only in an indicative manner. Tests that have already been standardized do not cover the full range of needs—namely, the assessment of all levels of comprehension across all age groups. The developed set of test stimuli enables continuous measurement of comprehension in Romani at the level of single words, word combinations, or entire sentences, from low difficulty (general comprehension sentences) to high difficulty (reversible sentences, passive constructions, participles, etc.).

For assessing comprehension in very young children, the Test of Communicative Behavior (Test komunikačného správania—TEKOS) [4] is used. It is standardized in Slovakia and designed for children up to 3 years of age. The test is administered when a speech or language delay is suspected; it takes the form of a questionnaire completed by the parent, who evaluates the child’s communicative abilities. Another tool used in this age group is the Munich Functional Developmental Diagnostics, which includes eight subtests, one of which assesses the child’s comprehension of speech by observing paralinguistic, lexical-semantic, and grammatical-syntactic understanding [5]. In preschool children, word-level comprehension is assessed, e.g., using the Crosslinguistic Lexical Task (CLT), which is designed for children aged 3–7. The test consists of 64 nouns and verbs that the child is asked to identify [6,7].

Sentence-level comprehension is evaluated, e.g., with the Drawing Test of School Readiness, in which the child draws shapes and figures according to verbal instructions. This test also provides insight into memory, motor coordination, and visual abilities [8]. Another assessment tool is the Test of Auditory Comprehension (APOR), which includes both closed tasks (selecting the correct picture) and open tasks (answering questions, repeating sentences, or performing a given instruction) [3].

In school-aged children, comprehension is most often assessed using tools primarily designed to evaluate academic literacy. Sentence-level comprehension may be examined through tasks such as reading discrimination tests or cloze reading tests (reading test with word completion) [7]. Another test used in this age group is the TROG-2 (Test for Reception of Grammar), which has been adapted into Slovak. This test consists of blocks targeting different grammatical structures [9]. The child selects the correct representation of a sentence from four images, three of which serve as distractors. Text-level comprehension is assessed using the Test of Reading Comprehension, which includes two stories read aloud by the examiner. The child then chooses the correct answer from four picture options, with questions addressing both explicit and implicit information [10]. In Europe, the ROMLAT test is also available, which is focused on grammar: syntax and morphology, rather than vocabulary [11]. It assesses children’s language competencies using various types of tasks.

For the assessment of adult patients, several diagnostic tools are used, including Picture Naming and Immediate Recall Test—PICNIR (Test pomenovania obrázkov a ich vybavenie—POBAV) [12], sentence comprehension tests [13], and the Boston Diagnostic Aphasia Examination [14,15]. These tests enable clinicians to evaluate lexical retrieval, syntactic processing, and broader aspects of language comprehension in adults.

At this stage, we have not conducted research that would statistically prove that Romani tests are better than Slovak tests for Roma. However, we are based on experience from visiting schools and preschools, where insufficient command of the Slovak language is reflected in the poorer outcomes for children. Examining the hearing or speech comprehension of Romani children through the Slovak language, which they do not sufficiently master, will unnecessarily worsen the test result and does not reflect the real needs of the child. Similarly, work focused on language barriers in healthcare suggests the importance of using the language that patients speak best [16]. With created material, we offer specialists the opportunity to perform speech audiometry in the Romani language, which will remove the language barrier and increase the effectiveness of the examination and the relevance of the results achieved.

## 3. Methods

This section describes the methodological procedures used to create the Roma speech dataset. The goal was to develop linguistically and culturally appropriate speech materials based on natural Romani language use.

### 3.1. Development of the Roma Speech Dataset

The Roma speech dataset was developed with the assistance of Romani-speaking individuals, including both children and adults, who indicated whether they were familiar with a given word in Romani and, where applicable, provided alternative lexical variants. The research was conducted across several locations in eastern Slovakia, specifically Bardejov, Jarovnice, Košice–Luník IX, and Rožňava [17].

**Figure 1:**
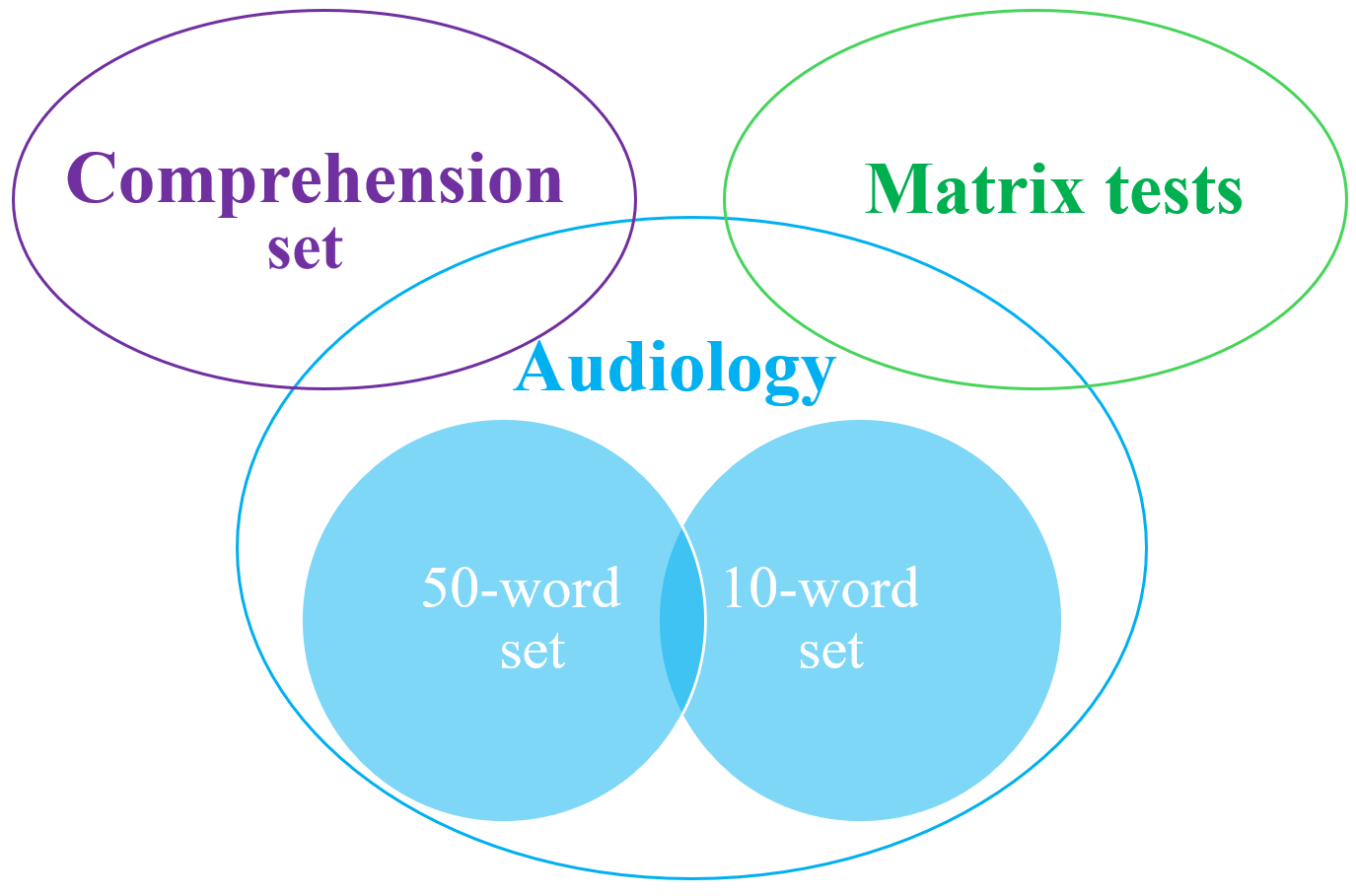
Composition of the Roma speech dataset.

The presented Roma speech dataset (Figure 1) contains sound data for:

- Audiology;
- Matrix tests;
- Comprehension.

### 3.2. Recording Procedures

The recording of speech stimuli for the Roma speech dataset was carried out in a soundproof room equipped with professional recording equipment. During the recording of the speech signal, emphasis was placed on capturing its natural acoustic form without the application of any additional sound processing, such as audio effects or equalization functions. Such modifications could significantly alter the frequency spectrum of speech, thereby distorting the natural characteristics of the voice, including formant structure, the intensity of individual phonemes, and subtle dynamic differences in articulation. Interventions of this kind could negatively affect the acoustic properties of the signal and lead to biased or misleading conclusions in diagnostic assessments. Recording and editing were performed in Adobe Audition.

All recordings are provided as single-channel audio without added noise and are normalized to a uniform loudness level, but in the case of the matrix test, the material is available in stereo format, with the speech signal in one channel and babble noise in the other, allowing flexible use in various SNR conditions.

Recording of stimuli was performed sequentially, with a larger time span between individual recording sessions. All recordings for the Roma speech dataset were produced using a consistent voice. The speaker was a native Roma woman who uses the language actively in everyday communication. She also participated in verifying the linguistic accuracy of the stimuli and contributed substantially to the design and translation of the items into Romani. Her collaboration in developing the Roma speech dataset was financially compensated.

### 3.3. Participants and Linguistic Validation

Special attention was devoted to the audiological part of the Roma speech dataset [17,18]. A total of 50 children participated in verifying the stimuli. Of these, 27 children attended kindergarten (aged 3–5 years, mean age 4 years) and 23 children attended primary school (aged 8–16 years, mean age 12 years). The children in kindergarten primarily used their mother tongue; however, they gradually began acquiring the Slovak language through regular educational activities and communication with their teachers, who supported their preparation for entering primary school. In addition, all stimuli in the Roma speech dataset were reviewed by three adult speakers over the age of 35, who are fluent in both Romani and Slovak. This additional verification ensured linguistic accuracy, naturalness, and cultural appropriateness of the presented materials.

### 3.4. Principles for Selecting Words and Sentences in the Roma Speech Dataset

The following subsections describe how these principles were applied in developing the audiology materials, the matrix test, and the comprehension dataset.

#### 3.4.1. Audiology

The Audiology section of Roma speech dataset contains words and multi-word expressions intended primarily for the assessment of auditory abilities. It is inspired by children’s vocabulary and includes items that children from socially disadvantaged backgrounds may encounter. These environments are often characterized by limited access to stimuli, material resources, and linguistically enriching input. For this reason, the database also incorporates high-frequency expressions from everyday life that reflect the typical life of children growing up under conditions of social disadvantage, while ensuring that the test set remains both culturally and linguistically relevant for the target group [17,18].

Mental processes in children up to the age of two are grounded in their direct experiences—what they see, hear, and touch. This developmental period corresponds to sensorimotor thinking, characterized by practical intelligence [19]. In preschool and early school age (2–7 years), visual–concrete thinking develops, which is based on images. During this period, the child uses available stimuli from the environment, but also from illustrations and from listening to stories. This leads to comparing information and drawing simple inferences. To foster this stage of reasoning, both real objects and visual materials such as pictures and illustrations should be used as starting points for commenting and interpreting the surrounding reality [20].

The 50-word set was directly designed for children [21]. The selection of these items was guided not only by linguistic and phonetic considerations, but also by developmental factors that determine how children at different ages perceive, process, and learn new lexical items [22]. Understanding these developmental characteristics is essential for ensuring that the chosen words are appropriate, comprehensible, and usable in clinical and diagnostic contexts.

The 10-word set was designed for rapid hearing screening. The selection criteria included word familiarity, the syllable length of the word, and the requirement for an unambiguous visual representation of each item.

The database is constantly expanding with new stimuli gradually added to the existing word set. The ongoing expansion of the database is motivated by several factors, including the need to enrich the list with new stimulus categories and to include words relevant to clinical practice based on feedback from audiologists, educators, and parents. Continuous updates also ensure that the database remains developmentally appropriate for different age groups and adaptable to emerging diagnostic needs in audiology.

#### 3.4.2. Construction of the Matrix Test

The first matrix test was developed by Hagerman [23]. The test is based on the principle of combining words contained in a 5 × 10 matrix. The matrix consists of five columns (categories), representing the subject, verb, numeral, adjective, and object. Each category contains ten words, and when constructing a sentence, one word is selected from each category. In this way, five-word sentences are generated, each following a fixed grammatical structure while collectively incorporating all 50 words. A single test then comprises ten such sentences [24].

From the perspective of the adaptive matrix test design, selecting suitable words that will form meaningful sentences in any possible combination is a demanding task, particularly when linguistic criteria such as word length and a comparable representation of phonemes characteristic of the language should be preserved. Nevertheless, the most essential criterion remains the familiarity of the words to the broadest possible audience because it is difficult to objectively assess the degree of deficit based on linguistically incompatible data [16].

Due to their difficulty (recognizing all the words in a sentence against a background noise), matrix tests are intended primarily for adults.

#### 3.4.3. Development of the Comprehension Dataset

General comprehension sentences play an important role in comprehension testing because they assess not only a child’s linguistic abilities but also the level of their encyclopedic knowledge and conceptual understanding of the world. This type of sentence requires the child to connect the linguistic input with long-term memory, everyday experiences, and the ability to form categories (animals, colors, means of transport, body functions). Successful performance on such items indicates that the child can adequately understand sentences commonly encountered in communication and that they grasp the basic relationships appropriate for their developmental stage [20,22]. These sentences also serve as a sensitive indicator of cognitive development: the child must work with more abstract concepts, select relevant information, and disregard irrelevant details. As a result, they provide valuable diagnostic insights into both language comprehension and the child’s ability to acquire new information.

Descriptive stative sentences represent an important category of linguistic constructions that are highly suitable for assessing language comprehension across various age groups and developmental profiles [25]. Their core characteristic is that they do not express an action but rather the current state, property, quality, or spatial arrangement of an object or phenomenon. Sentences such as “the books are arranged on the table”, “the carpet is dirty”, or “the lunch is completely eaten” allow specialists to observe how accurately and efficiently an individual can construct a mental representation of a situation based on static semantic information. From the perspective of linguistic processing, these sentences are significant because they often contain adjectives or past participles (e.g., arranged, locked, folded), which require a precise understanding of the resulting state. Another important aspect is their relative temporal neutrality. Since they do not involve event sequencing, processing them does not burden working memory with temporal analysis. This allows for a more precise isolation of pure semantic comprehension without interference from aspectual or temporal relations. Furthermore, stative sentences are easily linked to visual stimuli, making them a highly effective tool for diagnostics in children with communication disorders, language delay, or specific learning difficulties [22].

Negative sentences have a specific diagnostic value in comprehension testing because they require more than simple recognition of words and objects—they assess the logical processing of meaning. Understanding negation presupposes that the child (or adult) first activates a positive mental representation of the situation and then suppresses or rejects it. This two-step process places increased demands on working memory, inhibition, and executive functions [26]. Negative sentences are important because they reveal the difference between surface-level comprehension (recognizing familiar words) and deep semantic comprehension (correctly interpreting the truth conditions of a sentence). In comprehension testing, minimal pairs are often used (e.g., “the apple is on the table” vs. “the apple is not on the table”), as they allow for precise identification of whether the individual processes the meaning of the sentence as a whole. In children, in particular, negation reliably reflects developmental levels of language comprehension, since mastery of negation emerges later than the understanding of affirmative statements and is sensitive to language disorders.

Spatial sentences play a crucial role in comprehension because they link language to the perception of space and to the mental representation of relationships between objects. They enable the speaker to specify the precise location of entities and allow the listener to correctly interpret spatial relations (inside, next to, on, in front of, behind), not merely the objects themselves. Understanding such sentences requires the integration of lexical meaning, grammatical structure, and visuospatial abilities, making them also a sensitive indicator of both linguistic and cognitive development. Sentences involving left–right orientation occupy a special place in the assessment of children’s comprehension. Their correct interpretation requires perspective alignment; that is, the child must determine from whose point of view the spatial orientation is being evaluated. This imposes higher demands on cognition, working memory, and spatial perception, reliably revealing subtle comprehension difficulties that may remain unnoticed in simpler spatial relations [27].

Passive sentences illustrate passive constructions as a universal linguistic means of shifting the perspective on an event. The passive voice allows attention to be redirected from the agent (the performer of the action) to the patient or to the event itself, while the agent may be omitted, left unspecified, or expressed explicitly. The significance of these sentences lies in the fact that they assess the ability to identify semantic roles independently of the surface structure of the sentence. They test whether the listener correctly understands who is acting, what is happening, and to whom the event applies, even when such information is not expressed directly or is secondary from the sentence’s perspective [28].

Reversible sentences illustrate the phenomenon of semantic reversibility, that is, the possibility of exchanging the roles of agent and patient without violating grammaticality or semantic acceptability [29]. In reversible verb constructions, both participants can function either as the initiator or the recipient of the action, and switching their roles leads to a different interpretation of the situation.

Sentences for testing participles illustrate the fundamental distinction between eventive (process-oriented) and stative (resultative) expressions. Eventive forms describe an ongoing action or a change unfolding over time—the focus is placed on the process itself (e.g., something is burning, flowing, or falling). Stative forms, by contrast, denote the outcome of a completed event and describe a static condition without internal temporal dynamics (e.g., something is burnt, spilled, or fallen). Understanding eventivity is important because it shapes the interpretation of time, causality, and visual meaning: it determines whether a situation is perceived as an ongoing event or as an already established state, which is crucial for accurate comprehension of the described scenario [30].

Sentences involving number contrasts (singular–plural) play a crucial role in comprehension assessment, as they test the ability to integrate grammatical information with a quantitative representation of reality. Understanding such sentences requires not only recognizing the object or action but also correctly interpreting how many participants or items are present in the situation. For children, number-contrast sentences are developmentally important: they reveal whether the child interprets grammatical number as a meaning-bearing category rather than merely a formal pattern [31]. For this reason, they are frequently used in language comprehension diagnostics and in identifying specific language difficulties.

Together, these categories enable a graded assessment of comprehension, from basic semantic processing to advanced morphosyntactic interpretation.

### 3.5. Development of Visual Stimuli

The use of visual support enables the child to understand the meaning of words more easily [32]. In children with hearing impairment, the process of learning new words unfolds gradually. It progresses from understanding the meaning of a word or expression (e.g., the child hears the word house while looking at the house they live in) to the ability to recognize its graphic equivalent (e.g., pointing to an illustration representing a house) [20].

The unambiguous visual representation [32] ensures that children are not limited in their mode of response and may choose whether to point to a picture, repeat the word, or write down the stimulus they hear. The picture-pointing method is particularly advantageous for young children and for patients with speech-production difficulties, as it eliminates the demand for verbal output. Moreover, the use of pictures is closely linked to comprehension, thereby supporting all stages of auditory skill development: detection, discrimination, identification, comprehension, auditory attention, and auditory memory [8].

The 50-word set (comprising both single words and multi-word expressions) is supported by a set of 50 illustrative picture cards designed to facilitate comprehension, especially for young children and early learners. These visual materials were created by a professional graphic designer who closely followed a set of predefined criteria to ensure that the illustrations are developmentally appropriate, visually clear, and culturally accessible. The visual design principles applied during the creation process included the following:

- A simple, child-friendly drawing style, ensuring that the depicted objects and actions are instantly recognizable even for children with limited vocabulary or developing perceptual skills;
- The elimination of unnecessary details and shadows, which reduces visual noise and prevents potential misinterpretation of the target motif;
- A limited color range with the use of a consistent color palette, supporting visual coherence across the entire set and minimizing distractions that might arise from overly vivid or contrasting colors.

Together, these design criteria contribute to the creation of visual stimuli that are not only esthetically unified but also functionally effective for diagnostic and educational purposes. The picture cards thus help bridge auditory input and visual recognition, allowing clinicians to assess comprehension, attention, and categorization skills in a controlled and child-appropriate manner.

**Figure 2:**
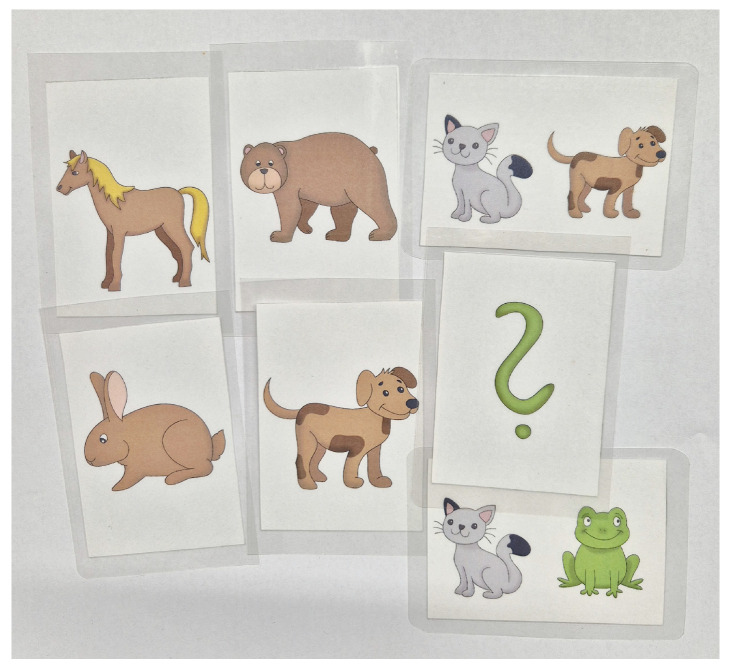
The example of picture cards.

The images are provided in JPG format, with the target motif displayed prominently in the foreground on a plain white background (see Figure 2). Hand-drawn illustrations are prepared in format: 1242 × 1754, 150 PPI, ensuring sufficient clarity for use in diagnostic settings and for accurate visual recognition by children.

For the set of 10 disyllabic words, the images were generated using AI tools. The same requirements were applied to the AI-generated images as to the hand-drawn ones. However, the images look more realistic and are more elaborate. Their generation was preceded by research described in the work [18].

### 3.6. Statistical Analysis

This study does not include inferential statistical testing, as no behavioral or audiological performance data were collected. The analysis is therefore limited to descriptive characterization of the speech materials (e.g., phoneme distribution and structural properties), which aligns with the developmental nature of this work. Inferential statistics, normality testing, and power calculations will be applied in future clinical validation studies.

## 4. Results

The presented Roma speech dataset spans several levels of complexity—from simple word-level stimuli designed for rapid screening (a set of ten two-syllable words), through a more extensive 50-word set with visual support tailored especially for children, up to an adaptive matrix test, which enables the assessment of hearing limits even under noisy listening conditions. In addition, a comprehension dataset was created to evaluate the processing of more complex spoken information, which focuses on assessing higher-level language processes that go beyond mere auditory detection.

At present, the Roma speech dataset includes the following sets within the audiology section: (1) a general set (150 stimuli/2 min 14 s); (2) the 50-word set (80 stimuli/1 min 31 s); (3) the 10-word set (10 stimuli/10 s). The Section 4.3 contains 300 stimuli (21 min 52 s) and a 1 kHz calibration tone (1 min). The Section 4.5 currently includes 213 stimuli (approx. 10 min), with at least 20 items available in each category. The data are not divided into training and testing subsets.

### 4.1. A 50-Word Set for Children

The words contained in this set are nouns denoting, for example, means of transport, food, parts of the human body, animals, toys/objects, and colors.

**Figure 3:**
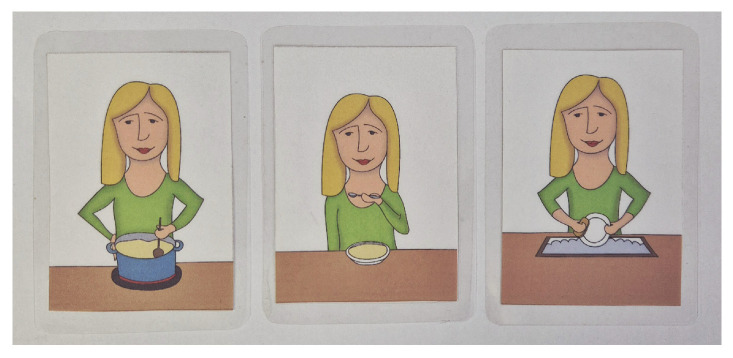
Example of picture cards from the combination category.

The set also includes a “combination” category, which is considered more demanding in terms of comprehension. This is because the corresponding picture cards are visually more complex, and the associated auditory stimulus conveys more specific information [33]. For example, the mother is cooking soup, the mother is eating soup, and the mother is washing the dishes (Figure 3). All illustrations depict the mother, but each shows her performing a different action. For the child to identify the correct picture, they must not only hear the stimulus but also correctly understand and distinguish the action being described, with the crucial information placed at the end of the sentence.

With the help of these stimuli, it is also possible to test the sequence of events (first the food is cooked, then the food is eaten, and finally the cleaning is performed). This requires the child to understand the entire context of the visualized event. A different situation occurs with the combination of picture cards featuring a blue, green, and red car. These stimuli consist of two-word expressions where, in contrast, the essential information appears at the beginning of the auditory stimulus. The inclusion of this category makes it possible to examine more complex cognitive processes, such as short-term memory, attention, knowledge of actions, colors, and similar abilities. The child may be presented with stimuli drawn from a single category or from multiple categories.

Upon recommendations from both parents and clinicians, the presented set of stimuli was expanded to include diminutive forms of selected words. This applies primarily to the categories of animals, food, and toys/objects. In total, 23 nouns are offered with an alternative diminutive form.

**Figure 4:**
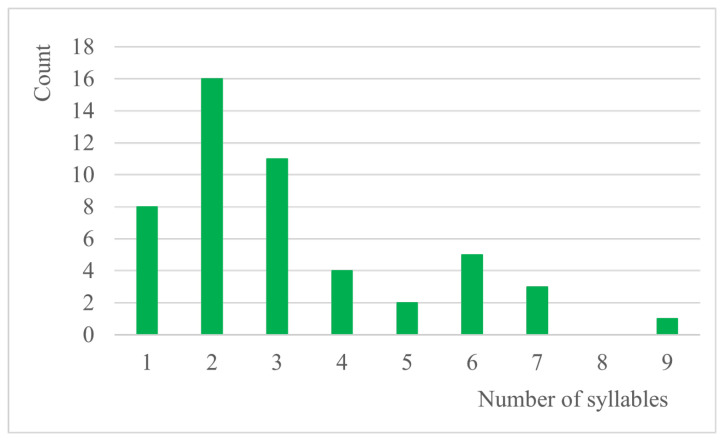
Syllable length of words.

The average length of the stimulus is 3.18 syllables (excluding the initial article o/e that precedes each item). The distribution of words according to their syllable length is presented in Figure 4. When diminutive forms are used, the average syllabic length of the speech stimulus increases by 0.58 syllables.

The selected words allow perception tests to be adapted to the child’s age and hearing abilities. The selection of words took into account the cultural and linguistic environment in which Romani children grow up.

Nevertheless, we consider it appropriate for parents to review the words prior to testing to ensure that they are familiar and suitable for the child.

### 4.2. A 10-Word Set

For screening purposes, ten two-syllable words were identified that have all the necessary characteristics to fulfill this objective effectively. These words are well known to children, and their uniform syllabic length ensures a comparable level of perceptual difficulty. Within the set of ten words, the phonemes present include sounds characteristic of the Romani language, as well as those that, through their frequency properties, cover the spectrum of the speech signal from low frequencies (*m*, *u*) through mid frequencies (*i*, *a*) to high frequencies represented by *s* or *š* [34]. Although the phoneme *š* is not included in the proposed list, it is functionally substituted by the phoneme *č*, which exhibits a similar noise-based spectral structure, differing primarily by the presence of a brief stop closure preceding the fricative release.

The list of 10 disyllabic Romani words consists of: table—o skamind, bread—o maro, bowl—o čaro, apple—e phabaj, window—e blaka, leg—o pindro, doll—e popka, frog—e žamba, cat—e mačka, castle—o burkos. Each stimulus is accompanied by an image.

### 4.3. Matrix Test

Between 2019 and 2021, research and development were conducted to create speech tests based on an adaptive matrix in the Romani language for Romani speakers living in Slovakia [35,36]. Word design, matrix creation, recording, editing and sentence composition were carried out in the LICOLAB at the Faculty of Arts, Pavol Jozef Šafárik University in Košice. The resulting matrix (Table 1) allows for the generation of up to 100,000 grammatically correct and semantically plausible sentences, ensuring high variability while maintaining controlled linguistic structure. The basic collection of 100 sentences includes all inter-word transients. A total of 300 sentences are available.

An essential component of matrix tests is the noise, which is generated from the recordings used within the test. It is created by mixing the individual sentence recordings in such a way that no distinct speech sounds can be recognized. The resulting noise, therefore, exhibits a spectral composition similar to that of the presented speech stimuli. Due to the close similarity between the noise spectrum and the spectrum of the target signal, this type of noise is considered highly challenging for the listener.

### 4.4. Acoustic and Linguistic Characteristics

In the previous sections, individual parts of the database were introduced. However, an overall view of the phonemic content of the collected data is available in this section. It should be noted that Romani is presented in contrast to Slovak, as Slovak—the dominant language in our country (Slovakia)—can significantly influence its use.

The standardized Romani alphabet comprises five vowels and twenty-nine consonants: *a*, *b*, *c*, *č*, *d*, *ď*, *dž*, *e*, *f*, *g*, *h*, *ch*, *i*, *j*, *k*, *kh*, *l*, *ľ*, *m*, *n*, *ň*, *o*, *p*, *ph*, *r*, *s*, *š*, *t*, *th*, *ť*, *u*, *v*, *z*, *zh*. Unlike Slovak, Romani does not have diphthongs and does not use *y*, but it does feature a set of soft consonants—*ď*, *ť*, *ň*, and *ľ*—marked orthographically with a caron. The language also contains several consonant pairs, such as *č–čh, k–kh, t–th*, as well as *ph* and *th*, which are pronounced with a light aspiration. These phonemic contrasts are meaningful: for instance, khoro (jug) differs from koro (blind) solely by aspiration. Polysemy is likewise common; for example, the verb anel may carry the meanings ‘to bring’, ‘to carry’, or ‘to fetch’ depending on context. A further distinction from Slovak lies in the grammatical system, which recognizes eleven parts of speech (all Slovak ones plus the definite article), uses two genders (masculine, feminine), one type of declension, and one type of conjugation, with no declension or verb paradigms, etc. [37,38].

**Figure 5:**
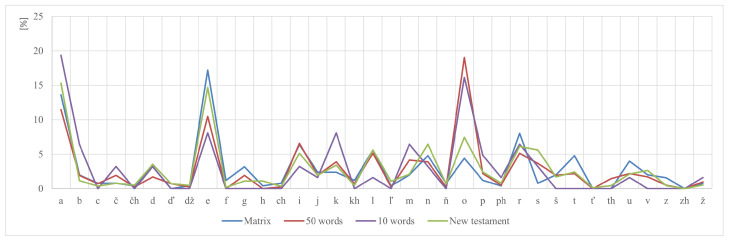
Comparison of phoneme distribution in the adaptive test matrix, 50-word set, 10-word set, and in the New Testament.

In the following picture (Figure 5), it is possible to see phoneme distribution across the proposed datasets.

The distributions of proposed sets were computed at the phoneme level, as well as Romani digraphs (*kh*, *ph*, *th*, *ch*, *zh*, *dž*), which were treated as single phonemes. As a natural language baseline, we used a Romani New Testament (Evaňjelium le Marekoskro) [39]. Across all datasets, the three most frequent categories are vowels *a*, *o*, *e*. Consonants *r*, *n*, *l*, *k* show higher occurrence, while aspirated pairs (*čh*, *kh*, *ph*, *th*) and soft consonants (*ď*, *ť*, *ň*, *ľ*) occur less frequently, which is expected given their lower lexical frequency in everyday vocabulary.

From an audiological perspective, the inventories cover phonemes mapping to the Ling six sounds (low: *m*, *u*; mid: *i*, *a*; high: *s*, *š* or the affricate *č* as a functional substitute for *š* in case of 10-word subset), ensuring that stimuli span the key frequency regions relevant for speech detection, discrimination, and identification.

Phoneme distribution analysis shows that the Matrix test exhibits very high similarity to the natural language reference (r = 0.92; cosine = 0.95), indicating strong phonological representativeness and supporting its suitability for realistic assessment of speech in noise.

The 50-word set shows good convergence with the reference corpus (r = 0.80, cosine = 0.88), while the 10-word screening list naturally diverges more (r = 0.78, cosine = 0.85) due to its minimal and purpose-oriented design. These results confirm that all three sets capture the core phonological structure of Romani to an appropriate degree for their intended clinical use.

This supports the intended diagnostic use: the 50-word set provides balanced pediatric material with pictorial support; the 10-word subset serves as a rapid screening tool; and the Matrix test offers dense phonological coverage suitable for testing in noise.

### 4.5. Comprehension Set

Comprehension testing is performed to find out the level of morphological and syntactic comprehension. The tested person needs to correctly decode the grammatical and semantic meaning of a sentence. The proposed Romani set is derived from a Slovak set of sentences for comprehension testing [40], but not all sentence structures that are grammatically correct in Slovak are also used in Romani. The services of a native speaker were used to assess the correctness of individual sentences. The comprehension set includes eight typical sentence categories:

- General comprehension sentences
  Proposed sentences are concerned with colors (“Savi farba hin pro kham?”—What is the color of the sun?), toys and everyday objects (“So penge o čhave šaj čhivkeren?”—What can children throw?), means of transport (“Savo dromeskero verdan džal pal e koľeja?”—Which means of transport runs on rails?), animals (“Savo džviros džanel te hurňaľol?”—Which animal can fly?), body parts (“Soha šaj šunas o hangi?”—What makes us hear sounds?), activities (“Sikav o čitro kaj e džuvľi tavel.”—Select a picture in which a woman is cooking.) and emotions (“Sikav o čitro pre savo hino o žalosno muršoro.”—Select a picture with a sad boy).
  Answers to sentences of this type should be mastered by children of preschool age, as they encounter them during play, getting to know the world, and preparing for school attendance.
- Descriptive stative sentences
  In comprehension assessments, this makes it possible to capture subtle differences in semantic and morphological competence, since the child/adult must distinguish contrasts such as clean–dirty, open–closed, calm–rough, or warm–cold. For example, the sentence “The crayons are broken.” tests whether the individual understands a qualitative change in the object or the resulting arrangement of the same object “The crayons are scattered.” or “The crayons are arranged.”
- Negative sentences
  The database contains negative sentences expressed through the negation “na”, which is placed before the verb, for example: “O rikono na bešel pre phuv.”—The dog is not sitting on the ground. In the third person singular and plural, the negative form of the verb to be (te jel)—“nane”—is used, for example: “Pro savo čitro nane o čhave?”—On which picture are the children not present? Both forms of negation are used in the database.
- Spatial sentences
  The spatial sentences included in the database assess the child’s ability to understand spatial orientation through the use of prepositions. The prepositions employed are commonly used ones: in, on, next to, in front of, under, at, and behind. Sentences including adverbs of place left and right were added into this category (“Pro savo čitro hiňi e žamba pre čačutňi sera?”—In which picture is the frog on the right?).
- Passive sentences
  Romani does not have commonly used passive sentence forms as, for example, English or even Slovak do. Passive constructions are relatively rare and are often formed using “pes”, as in: “Kide avri o čitro, pre savo pes thovkeren o grati.”—Select a picture in which dishes are washed.
- Reversible sentences
  These sentences show semantic reversibility, i.e., both participants can switch roles (agent–patient) without breaking grammar or meaning, resulting in a different interpretation, for example, “E pheň denaškerel pal o phral.”—Sister is chasing brother. or “Pal o phral denaškerel e pheň.”—Brother chases sister.
- Sentences for testing participles
  This type of sentence tests the understanding of participles by distinguishing between eventive and stative meanings. It highlights the difference between an ongoing process and the resulting state after its completion, for example, “Which picture shows a burning fire?”—“Pro savo čitro hin e jag savi labol?” versus “Which picture shows a burnt out fire?”—“Pro savo čitro hiňi e jag savi nalabol?”. This distinction is universal across languages, although it is expressed through different grammatical means.
- Sentences for testing count
  These sentences simultaneously assess several levels of processing: morphological analysis (noun number and agreement with the verb), the semantics of quantity, and visual discrimination between one versus multiple objects. Minimal pairs such as “The child is playing with cubes.”—“O čhavoro pes bavinel le kockenca.” versus “The children are playing with cubes.” or “O čhavore pen bavinen le kockenca.” or “There is a pear on the table.”—“Pro taňiris hine o ambrola.” versus “There are pears on the table”—“Pro taňiris hin e ambrol.” are highly sensitive, because the contrast is often signaled only by a subtle formal change.

## 5. Discussion

This work presents the first systematically developed set of Romani language speech materials intended for audiological and comprehension assessment. The datasets demonstrate balanced phonological coverage, reflect natural language use, and offer linguistically appropriate stimuli for both clinical and educational settings. Although clinical validation has not yet been conducted, the linguistic analyses presented here indicate that the materials meet the structural and phonetic requirements necessary for their intended diagnostic applications.

The mother tongue plays a fundamental role in thinking, meaning-making, and everyday communication. It provides the natural cognitive framework within which a child constructs initial mental representations of the real world, acquires concepts, forms coherent ideas, and on this basis develops core linguistic structures. When Romani-speaking children in Slovakia enter preschool, they are systematically confronted with the state language (Slovak), which for many of them constitutes a second language. Consequently, their academic success and social inclusion depend directly on their ability to communicate effectively in Slovak.

For children with communication disorders, this discrepancy is even greater: they struggle in both languages, but only the mother tongue allows an accurate evaluation of their real linguistic level [41,42]. Testing exclusively in Slovak may therefore misrepresent their capacities and delay the identification of genuine speech–language problems. As a result, many Romani children are unfairly judged as linguistically or cognitively weak. Early diagnosis, however, must rely on a language the patient (child/adult) understands. Romani-language diagnostic materials thus enable fairer, earlier, and more reliable identification of communication difficulties.

To our knowledge, no Romani-language materials suitable for speech audiometry are currently available in Slovakia. The resources developed in this study can be considered unique. They include three complementary components: (1) a set of audiology stimuli designed mainly for children, (2) an adaptive matrix test primarily intended for adult Romani speakers, and (3) a comprehension test covering multiple grammatical and semantic structures. Together, these datasets provide a foundation for conducting audiological and linguistic assessments in the patient’s mother tongue, thereby reducing the risk of misdiagnosis associated with language barriers.

All presented test materials represent linguistically and culturally appropriate stimuli usable in clinical practice for Romani-speaking children and adults. However, at this stage, the presented datasets have not been clinically validated; therefore, we do not claim diagnostic sensitivity, specificity, or normative performance curves.

In settings where no Romani speech materials exist, these datasets can already function as a practical tool for overcoming the language barrier and improving the validity of audiological examinations or comprehension assessment.

## 6. Conclusions

The Roma speech dataset fills a critical gap in audiological assessment and comprehension assessment of Romani-speaking populations in Slovakia by providing the first systematically developed set of speech materials in Romani for clinical and diagnostic use. In contrast to existing assessment tools available in Slovak, the proposed datasets are linguistically natural, culturally appropriate, and based on authentic language use by Romani-speaking children and adults.

The developed materials cover multiple levels of auditory and language processing, from rapid word-level screening and pediatric audiometry with visual support to advanced assessment of speech perception in noise using an adaptive matrix test and detailed assessment of morphological and syntactic comprehension. Together, these components form a comprehensive framework that allows professionals to assess hearing and speech comprehension in the patient’s native language, thereby reducing the risk of misdiagnosis caused by language barriers. From a clinical and educational perspective, the availability of Romani language stimuli represents an important step towards more equitable and accurate assessment of Romani-speaking children, especially in cases where insufficient knowledge of Slovak may mask actual language or auditory abilities. The use of assessment materials in the mother tongue allows for earlier identification of communication disorders and supports more informed decisions in subsequent intervention, therapy, or educational placement.

Although the presented datasets have not yet undergone clinical validation and normative testing, they already provide a valuable practical resource for audiologists, speech therapists, special educators, and researchers working with Romani-speaking populations. In settings where no diagnostic materials in Romani are available, the Roma speech dataset can serve as an effective tool to improve the relevance and interpretability of assessment results.

Future research will focus on clinical validation of the proposed tests and expanding the Roma speech dataset with new stimuli, including consideration of Romani dialects.

## Figures and Tables

**Table 1:** Romani word matrix.

Subject	Verb	Numeral	Adjective	Object	Translation
O Gejza	dikhel	efta	buchle	gende	Gejza sees seven wide books
O Peter	garuvel	ochto	bare	čitrišagi	Peter hides eight large paintings
E Eva	cinel	deš	cikne	roja	Eva buys ten small spoons
O Martin	ľidžel	eňa	phure	gereki	Martin wears nine old coats
E Helena	rodel	but	neve	pira	Helena looks for a lot of new pots
E Vjera	arakhel	frima	lačhe	lavuti	Viera finds a few good violins
E Jana	kidel	buter	žuže	khosne	Jana takes more clean scarves
O Jozef	mangel	biš	šukar	gada	Jozef asks for twenty nice dresses
E Marija	del	panč	dragane	trasta	Marija gives five expensive irons
E Zuza	kamel	trin	tuňe	košara	Zuza wants three cheap baskets

## Data Availability

The data, recordings, and drawings from this study will be available by the corresponding author upon reasonable request.
